# A cross-sectional assessment of metabolic syndrome in HIV-infected people of low socio-economic status receiving antiretroviral therapy

**DOI:** 10.1186/s13098-015-0008-5

**Published:** 2015-03-07

**Authors:** Eduard Tiozzo, Janet Konefal, Sarah Adwan, Lynell A Martinez, Juan Villabona, Johanna Lopez, Stacy Cutrono, Syed Muhammad Ahsan Mehdi, Allan Rodriguez, Judi M Woolger, John E Lewis

**Affiliations:** Department of Psychiatry and Behavioral Sciences, University of Miami Miller School of Medicine, Clinical Research Building, 1120 NW 14th Street, Miami, FL 33136 USA; Department of Family Medicine and Community Health, University of Miami Miller School of Medicine, Miami, FL USA; Department of Kinesiology and Sport Sciences, University of Miami, Miami, FL USA; Department of Biology, Miami-Dade College, Miami, FL USA; Department of Dietetics, Florida International University, Miami, FL USA; Sylvester Comprehensive Cancer Center, University of Miami Miller School of Medicine, Miami, FL USA; Department of Medicine, University of Miami Miller School of Medicine, Miami, FL USA

**Keywords:** HIV, Metabolic syndrome, HbA1c, Sugar consumption, Low SES

## Abstract

**Background:**

Metabolic syndrome (MetS) is a constellation of symptoms used as a measure to identify patients at increased risk for cardiovascular disease, type 2 diabetes, and all-cause mortality. The results of prolonged life expectancy and cumulative toxic effects of antiretroviral therapy increase the chance that HIV can cause clinical abnormalities, including MetS.

**Methods:**

We evaluated 89 people living with HIV (PLWH; mean age 48 ± 7 years; mean duration of HIV infection 17 ± 12 years; 47% men; 66% African-American, 22% Hispanic, and 10% non-Hispanic white; and 84% unemployed) enrolled in a community-based exercise training and nutrition education program targeting individuals of low socio-economic status (SES). The prevalence of MetS characteristics and the factors associated with the presence of MetS were analyzed.

**Results:**

One in three (33%; 12 men and 17 women) PLWH met ATPIII criteria for MetS. In our cohort, MetS was driven by high waist circumference and elevated blood pressure. In addition, higher use of protease inhibitors, elevated hemoglobin A1c (HbA1c), greater self-reported daily caloric intake and consumption of carbohydrates, sugar, added sugar, and higher glycemic load were found among the individuals with MetS, compared to those without it. Elevated HbA1c and high total sugar consumption were the strongest predictors and accounted for 30% of the occurrence of MetS.

**Conclusions:**

The overall prevalence of MetS in our PLWH cohort receiving antiretroviral therapy is higher than previously reported in the general population and in other PLWH cohorts. Additional work is needed to determine whether MetS is a more disease dependent or lifestyle dependent condition in PLWH.

## Background

Metabolic syndrome (MetS) is a condition characterized by the clustering of well-documented cardiovascular disease (CVD) risk factors with various underlying pathologies. According to the National Cholesterol Education Program/Adult Treatment Panel III (NCEP ATPIII) criteria, MetS is defined by three or more of the following features: abdominal obesity, hypertriglyceridemia, low levels of high-density lipoprotein cholesterol (HDL-C), insulin resistance, and hypertension [1]. MetS comprises risk factors independently associated with type 2 diabetes and CVD [2]. Identifying individuals with MetS and implementing therapeutic lifestyle changes along with medications may halt the progression of these chronic diseases. According to the latest report utilizing the National Health and Nutrition Examination Survey (NHANES), the prevalence of MetS among US adults was 23% [3].

The success of antiretroviral therapy (ART) has led to a decline in morbidity and mortality in people living with HIV (PLWH) [4]. As a result of efficacious and more accessible treatment, HIV has become a manageable chronic disease. However, prolonged life expectancy with HIV, accompanied with sustained toxic exposure to ART, can cause clinically relevant abnormalities, including the features of MetS [5]. Studies report different rates and predominant characteristics of MetS in PLWH, compared to the healthy population. Most studies have found a prevalence of 11% to 26% in PLWH, with this population’s rate being comparable or slightly lower than in the general US population [4,6-9]. Conversely, a few older Italian studies have reported higher rates in PLWH, ranging from 33% to 45% [10-12]. In terms of MetS criteria, most commonly, PLWH have hypertriglyceridemia and low HDL-C levels, compared to abdominal obesity, low HDL-C levels, and hypertension in the general US population [13]. Similar to general health care guidelines, HIV treatment guidelines recommend screening patients for MetS to reduce the risk for CVD morbidity and mortality. Thus, the purpose of this study was to evaluate and report: (1) the prevalence of MetS and each of its five characteristics and (2) factors associated with the presence of MetS in PLWH receiving stable ART and who are of predominantly low socio-economic status (SES).

## Methods

### Participants

Ninety male and female PLWH were enrolled in a community program targeting individuals of low SES called “Healthy Living for Better Days” through referrals from the Adult Special Immunology Clinic at University of Miami Miller School of Medicine/Jackson Health System and other local HIV clinics. Program eligibility criteria included: (1) confirmed HIV infection as established by external laboratory reports, (2) men or women ≥18 years of age, and (3) ability to attend weekly exercise sessions at the UHealth Fitness and Wellness Center at the University of Miami Miller School of Medicine. Program exclusion criteria included: (1) any medical condition or situation for which exercise would be contraindicated and (2) pregnancy in women. The program was approved by the Institutional Review Board for human subjects research of the University of Miami. All participants signed informed consent and HIPAA forms before commencing the program.

### Design

“Healthy Living for Better Days” was a 12-month community-based exercise training and nutrition education program aimed to improve the cardiovascular health of PLWH. We began recruitment in March 2013 and closed enrollment in November 2013. The program consisted of supervised combined aerobic and resistance exercise conducted three times a week and nutrition education conducted once a week. Outcome variables were measured at baseline and months three, six, and twelve, and they included: (1) physical characteristics (body weight, body mass index [BMI], waist circumference [WC], and blood pressure [BP]), (2) physical fitness (estimated maximal oxygen consumption [VO_2max_] and one-repetition maximum [1-RM] for upper and lower body strength), (3) lipid profile (total cholesterol [T-Chol], low-density lipoprotein cholesterol [LDL-C], very-low density lipoprotein cholesterol [VLDL-C], HDL-C, and triglycerides [TGs]), (4) non-lipid biomarkers (hemoglobin A1c [HbA1c], fasting glucose [FG], insulin, and C-reactive protein [CRP]), and (5) dietary intake (Block Brief Food Frequency Questionnaire).

### Physical characteristics

Anthropometric measurements were obtained by trained investigators using standard techniques. Weight and height were recorded to the nearest 0.1 kg and 0.1 cm, respectively, to calculate BMI. Waist was measured in centimeters (cm) at the narrowest circumference halfway between the lowest rib and the iliac crest. Systolic BP (SBP) and diastolic BP (DBP) were measured by use of an automatic oscillometric device, Omron BP monitor. Three readings were made with the subjects seated after they had rested for 5 minutes. The average of the second and third readings was used in the analysis [14].

### Physical fitness

Cardiorespiratory fitness was measured using a Rockport One-Mile Fitness Walking Test [14]. The test was modified for use indoors at the UHealth Fitness and Wellness Center with participants performing the one-mile walk on a treadmill rather than on an outdoor track as quickly as possible, and they were allowed to modify the speed (mph) at their discretion throughout the test. Heart rate was measured for ten seconds immediately upon completion by palpating the radial artery. Age, gender, body weight, and walk time were also recorded and used in a regression equation to estimate VO_2max_.

Muscular strength was measured using the American College of Sports Medicine’s protocol for 1-RM testing [14]. Program participants completed a maximum of four trials of 10, 8, 6, and 3 repetitions with rest periods between 2 and 4 minutes between trials. The initial weight was selected within the subject’s perceived capacity (50-70% of capacity), and resistance was progressively increased until the participants reached their maximum. The final maximum weight lifted successfully one time for bench press (upper body muscular strength) and leg press (lower body muscular strength) was recorded as the 1-RM.

### Blood sampling

Participants were instructed to arrive between 9:00 AM and 10:00 AM in a fasted state for blood sampling. Chemistry and immunoassays were performed by an automated analyzer (Roche Cobas-6000; Roche Diagnostics, Indianapolis, IN) utilizing the manufacturer’s reagents and following the manufacturer’s instructions. Total cholesterol and TGs were determined in serum or plasma by enzymatic, colorimetric assays. Intra-assay and inter-assay coefficients of variation (CVs) were 0.7% and 1.8%, respectively, for T-Chol and 0.9% and 2.3%, respectively, for TGs. High-density lipoprotein cholesterol was measured using a third generation homogenous enzymatic colorimetric assay, and the intra-assay and inter-assay CVs were 0.6% and 1.9%, respectively. Low-density lipoprotein cholesterol was calculated using the Friedewald equation. Fasting glucose was measured by the hexokinase method with intra-assay and inter-assay CVs as 1.9% and 2.7%, respectively. C-reactive protein was quantified in serum by a high sensitivity latex-particle enhanced immunoturbidimetric assay with a detection limit of 0.1 mg/L with an intra-assay CV of 1.1% and inter-assay CV of 2.2%. Hemoglobin A1c in whole blood was measured by high-performance liquid chromatography using a fully automated analyzer (Variant II Hemoglobin Testing System, BioRad, Richmond CA), and intra-assay and inter-assay CVs were 1.7% and 2.0%, respectively.

### Questionnaires

The Block Brief Food Frequency Questionnaire is a dietary assessment tool containing 55 questions, and it takes 20–35 minutes to self-administer [15]. The questionnaire obtains both frequency and quantity of food intake. The analysis estimates daily intake of: total calories (kcal), protein (g), total fat (g), saturated fat (g), trans fat (g), carbohydrate (g), total sugar (including naturally occurring sugar and added sugar) (g), added sugar (sugar and syrup added to food and beverages during processing and preparation (g), fiber (g), fruit intake (cup equivalent), vegetable intake (cup equivalent), average daily glycemic load (GL; g), and average daily glycemic index (GI).

Additional lifestyle data were obtained, which included self-reported daily smoking (none or less than a half pack, a half to one pack, and more than one pack), coffee consumption (none or average cups per day), alcohol consumption (none or average drinks per week), and average sleep hours per night. Data were also collected for lipid-lowering, antihypertensive, and diabetes drugs, current ART, and missed days of taking ART in the previous 90 days or since the last assessment.

### Metabolic syndrome

Metabolic syndrome was defined using ATPIII criteria [1]. Three or more criteria had to be met for defining MetS: (1) fasting serum TGs (≥150 mg/dl), (2) abnormal WC (>102 cm for men and >88 cm for women), (3) low HDL-C level (<40 mg/dl for men and <50 mg/dl for women), (4) high BP (≥130/85 mm Hg), or (5) high FG level (≥110 mg/dl). Participants who reported being diagnosed with diabetes or who were receiving treatment for diabetes were classified as having a high FG level. The same criterion was used for high BP.

### Statistical analyses

Metabolic syndrome was examined in relation to standard and other risk factors. Frequency and descriptive statistics were calculated on all variables. Student’s *t* test was used to assess differences between two means, and chi square was used to examine the degree of association of categorical variables. Variables demonstrating a significant univariate relationship with MetS were included in a logistic regression to assess their independent effect on MetS diagnosis. The following variables were included in the multivariate analysis: protease inhibitors (PIs) use, HbA1c, FG, and the consumption of total carbohydrates, sugar, and added sugar. SPSS 22 for Windows (IBM, Inc., Chicago, IL) was used for statistical analyses, and α < 0.05 was considered significant.

## Results

One participant not receiving ART was excluded, leaving 89 PLWH (mean age 48 ± 7 years; mean duration of HIV infection 17 ± 12 years; 47% men; and 66% African-American, 22% Hispanic, and 10% non-Hispanic white) with available data on criteria of MetS at baseline for the final analysis. More than three-quarters (83%) of our participants were not employed at the time of enrollment.

Seven (8%) individuals had no features of MetS. One feature was seen in 28 (32%) individuals, two in 25 (28%), three in 19 (21%), four in 8 (9%), and all five in 2 (2%) individuals. Thus, one in three (33%; 12 men and 17 women) PLWH met ATPIII criteria for MetS. In our cohort, MetS was driven by high WC and elevated BP (Table 1).

**Table 1:** **Individual features of MetS of study participants**

**Variables**	**Overall**	**MetS**	**No MetS**
n (%)	89	29 (33)	60
Waist circumference >102 cm in men and >88 cm in women	63 (71)	28 (97)	35 (58)
Blood pressure ≥130/85 mm Hg (or use of antihypertensive medication)	46 (52)	25 (86)	6 (10)
Triglycerides ≥150 mg/dl	19 (21)	14 (48)	5 (8)
Glucose ≥110 mg/dl (or use of anti-diabetic medication)	19 (21)	9 (31)	7 (12)
HDL-C <40 mg/dl in men and <50 mg/dl in women	13 (15)	10 (35)	2 (3)

Data are n (%). *MetS* metabolic syndrome, *HDL-C* high-density lipoprotein cholesterol.

Participants with and without MetS were of similar age and duration of HIV infection. We did not observe any differences in the incidence of MetS based on sex, ethnicity, or employment status.

The use of PIs was significantly higher among the participants with MetS (Table 2). According to ATPIII criteria, one in two individuals (48%) taking PIs had MetS, while among 36 participants not receiving PIs, only one of six individuals (17%) had MetS (p = 0.003).

**Table 2:** **Characteristics of study participants with and without MetS**

	**MetS** **(n = ** **29)**	**No MetS** **(n = ** **60)**	**P value**
Age (years)	49 ± 4	48 ± 8	0.54
Sex (%)			
Male	12 (41)	29 (48)	0.35
Female	17 (59)	31 (52)	
Duration of HIV (years)	17 ± 7	18 ± 15	0.32
ART (%)			
PI	22 (78)	24 (44)	0.003
Non-PI	6 (21)	30 (55)	
Ethnicity (%)			0.41
Non-Hispanic White	1 (3)	9 (15)	
African-American	22 (76)	36 (60)	
Hispanic	6 (21)	15 (25)	
Employment (%)			0.11
Unemployed	26 (93)	48 (80)	
Employed (part or full time)	2 (7)	12 (20)	
Systolic BP (mmHg)	132 ± 13	122 ± 10	0.000
Diastolic BP (mmHg)	84 ± 9	78 ± 8	0.002
Body mass index (kg/m^2^)	31.8 ± 7.0	31.0 ± 8.2	0.64
Waist circumference (cm)	108 ± 15.0	105 ± 20	0.46
Waist-hip ratio	0.93 ± 0.07	0.92 ± 0.07	0.38
VO_2max_ (ml/kg/min)	26.5 ± 7.7	27.3 ± 8.4	0.76
1-RM upper body (lbs)	112 ± 49	117 ± 53	0.69
1-RM lower body (lbs)	209 ± 93	232 ± 71	0.24
Current coffee drinker (%)	20 (77)	47 (78)	0.34
Current alcohol drinker (%)	6 (21)	16 (27)	0.54
Current smoker (%)	9 (31)	23 (38)	0.50
Sleep (hours)	6.5 ± 1.7	7 ± 1.8	0.70

Data are means ± SD or n (%).

*MetS* metabolic syndrome, *ART* antiretroviral therapy, *PI* protease inhibitors, *BP* blood pressure, *VO*_*2max*_ maximal oxygen consumption, *1-RM* one repetition maximum.

Blood pressure, one of the features of MetS, was expectedly higher (+9% for both SBP and DBP) in those with MetS than in those without it. The same finding explains the higher use of antihypertensive medication among the participants with MetS, compared to those without MetS (43% vs. 18%, p = 0.02).

Although not statistically significant, MetS was associated with less favorable physical characteristics and physical fitness levels. Participants with MetS, compared to the ones without MetS, had greater BMI (31.8 vs. 31.0, p = 0.64) and WC (108 cm vs. 105 cm, p = 0.46) and lower estimated cardiorespiratory fitness (26.5 ml/kg/min vs. 27.3 ml/kg/min, p = 0.76) and strength levels (upper body: 112 lbs vs. 117 lbs, p = 0.69 and lower body: 209 lbs vs. 232 lbs, p = 0.24).

All biochemical markers of CVD risk (Table 3) were less favorable among the participants with MetS. The greatest difference between the ones with MetS and those without MetS was observed for HDL-C (46 vs. 53 mg/dl; p = 0.07). Similarly, all biochemical markers of diabetes (Table 3) were less favorable in the MetS group, with HbA1c being significantly higher in the same group compared to those without MetS (6.4 vs. 5.7%, p = 0.03; Table 3). Self-reported use of diabetes medication was more prevalent in those with MetS (25% vs. 6%, p = 0.01).

**Table 3:** **Cardiovascular and diabetes biochemical markers in PLWH with and without MetS**

	**MetS** **(n = ** **29)**	**No MetS** **(n = ** **60)**	**P value**
T-Chol (mg/dl)	184 ± 41	182 ± 36	0.83
LDL-C (mg/dl)	110 ± 31	107.34	0.71
HDL-C (mg/dl)	46 ± 15	53 ± 15	0.07
VLDL-C (mg/dl)	28 ± 17	22 ± 12	0.12
Non-HDL-C (mg/dl)	138 ± 35	130 ± 38	0.32
Total/HDL-C	4.2 ± 1.1	3.7 ± 1.3	0.09
TGs (mg/dl)	139 ± 84	112 ± 56	0.13
CRP (mg/L)	6.5 ± 8.3	5.3 ± 7.3	0.52
FG (mg/dl)	103 ± 51	91 ± 10	0.21
HbA1C (%; mmol/mol)*	6.4;46	5.7;39	0.03
Insulin (μU/mL)	17.3 ± 13.1	13.4 ± 7.6	0.15
Insulin resistance (HOMA)	4.3 ± 3.4	3.0 ± 1.9	0.09

Data are means ± SD. *SDs for HbA1C are 1.7 (MetS) and 0.6 (No MetS).

*PLWH* people living with HIV, *MetS* metabolic syndrome, *T-Chol* total cholesterol, *LDL-C* low-density lipoprotein cholesterol, *HDL-C* high-density lipoprotein cholesterol, *VLDL-C* very-low density lipoprotein cholesterol, *TGs* triglycerides, *CRP* C-reactive protein, *FG* fasting glucose, *HbA1C* hemoglobin *A1C*, *HOMA* homeostasis model assessment.

Daily dietary behavior (Table 4) reveals significantly higher total energy intake among the participants with MetS, compared to those without it (+27%, +634 Kcal, p = 0.05). The higher energy intake in the MetS group comes from the disparity in carbohydrates (+30%, +348 Kcal, p = 0.01), as the only macronutrient significantly different between the groups, while fat (+24%, +176 Kcal, p = 0.10) and protein (+24%, +92 Kcal, p = 0.08) were marginally different. Participants with MetS reported significantly higher daily consumption of sugar (+38%, +228 Kcal, p = 0.008) and sugar in the forms of sugar and syrup added to food and beverages (+40%, +180 Kcal, p = 0.007). The intake of fruit, vegetables, and fiber was lower than the national recommendations in both groups. Even though the MetS group had slightly higher intake of these food groups and nutrients, we did not observe a significant difference between the groups. Self-reported coffee, alcohol, and smoking consumption and the amount of daily sleep also did not differ between the groups.

**Table 4:** **Nutrient intake in PLWH with and without MetS**

	**MetS** **(n = ** **29)**	**No MetS** **(n = ** **60)**	**P value**
Total calories	2359 ± 1470	1725 ± 1043	0.05
Fat (g)	92 ± 65 (31)	70 ± 43 (32)	0.1
Saturated fat (g)	31 ± 23 (11)	23 ± 14 (11)	0.09
Trans fat (g)	7 ± 8	6 ± 7	0.49
Protein (g)	95 ± 61 (16)	72 ± 40 (17)	0.08
Carbohydrates (g)	291 ± 145 (49)	204 ± 136 (47)	0.01
Sugar (g)	151 ± 97 (26)	94 ± 68 (22)	0.008
Added sugar (g)	112 ± 89	67 ± 62	0.007
Servings of fruit (cups)	1.5 ± 1.08	1.2 ± 1.0	0.17
Servings of vegetables (cups)	1.6 ± 1.2	1.3 ± 1.0	0.31
Serving of fruit/vegetables (cups)	3.1 ± 2.0	2.5 ± 1.7	0.17
Fiber (g)	14 ± 10	11 ± 7	0.08
Glycemic load (g)	142 ± 87	100 ± 68	0.02
Glycemic index	52 ± 5	52 ± 4	0.66

Data are means ± SD (% of total calories).

*PLWH* people living with HIV, *MetS* metabolic syndrome.

We conducted a multivariate logistic regression to determine which of the aforementioned significant variables (PIs, HbA1c, total sugar, added sugar, and GL) would predict the occurrence of MetS. The overall model was significant, as *X*^2^(2) = 18.8, p < 0.001, −2 Log Likelihood = 83.53, Nagalkerke R^2^ = 0.29, and 75% of the cases were predicted correctly. A 2.4 unit increase in HbA1c resulted in a greater likelihood of the presence of MetS (95% confidence interval [CI]: 1.2, 4.9, p = 0.02). While total sugar was significant (p = 0.01), the odds ratios of just 1.01 showed little change in this variable predicting the presence of MetS (95% CI: 1.0, 1.1).

## Discussion

Our cross-sectional analysis is part of a longitudinal program involving PLWH of predominantly low SES. Using ATPIII criteria, 33% of PLWH taking ART were estimated to have MetS at the time of their enrollment into “Healthy Living for Better Days,” an exercise training and nutrition education community program. The presence of MetS in almost one in three individuals was driven by a high rate of abdominal obesity and hypertension. Higher use of PIs, elevated BP and HbA1c, greater self-reported daily caloric intake and consumption of carbohydrates, sugar, and added sugar, and higher GL were recorded among the individuals with MetS, compared to those without it. Elevated HbA1c and high total sugar consumption were the strongest predictors and accounted for 30% of the occurrence of MetS.

Different definitions of MetS have been utilized to examine its incidence, and most studies have reported a rate of 11% to 26% among PLWH. The same rates have been comparable or slightly less in HIV-negative individuals. For example, the incidence of MetS in the NHANES dataset among adults 20 years of age and older was estimated to be 23% [3]. Overall, our PLWH cohort of 20 years of age and older had higher rates of MetS than previously reported in the general population. However, most of our participants were 40 to 59 years old, and their age-specific incidence of MetS was similar to the NHANES cohort (37% vs. 41% for males and 37% vs. 37% for females) [16]. It is still unclear if HIV infection per se or ART use has a dominant mechanistic role in the development of MetS. Similarly to one cohort from the northeastern US, we found no association between duration of HIV infection and MetS [7], while the information on duration of ART use among our participants was not available at baseline. However, it is worth noting that all of our subjects were receiving ART, with the overall prevalence of MetS in our cohort being higher than in other PLWH cohorts. The same rate is comparable (from 31% to 45%) to three younger PLWH Italian cohorts, where all study participants also had ART exposure [10-12].

The characteristics of MetS have been reported to differ between PLWH and the general population, and these differences could be attributed to HIV and/or its associated therapies. The most commonly achieved metabolic criteria for MetS in PLWH were hypertriglyceridemia and low HDL-C, while the least common criterion was increased WC [13]. The opposite was true for our cohort. The rates of hypertriglyceridemia and low HDL-C were among the lowest in our study. In regard to increased WC, HIV-associated lipoatrophy and loss of abdominal subcutaneous fat can actually lead to lower rates of central obesity [13]. Nonetheless, overall three out of four individuals and almost all individuals with MetS had high WC, making this trait the most common metabolic abnormality among our participants. The second most prevalent component of MetS in our study was hypertension. No specific ART has been associated with elevated BP, and underlying factors associated with hypertension, similar to the general population, are BMI and age [13]. The higher rate of obesity in our cohort could explain the increased rate of hypertension, both compared to the general population. Our findings are similar to the NHANES dataset [3], where the rates of abdominal obesity and hypertension, in addition to low HDL-C, were the most common features contributing to MetS. Therefore, we conclude that the overall incidence of MetS and the rates of its individual traits in our PLWH cohort resemble more the general population, rather than other PLWH cohorts.

Specific ART medications are known to affect individual components of MetS, and PI use is independently associated with metabolic abnormalities [17]. Protease inhibitors, a class of ART, are hypothesized to cause peripheral lipodystrophy, a syndrome that can lead to central fat accumulation, hyperlipidemia, and insulin resistance [18]. In our analysis, PI exposure was not an independent risk factor for MetS, but we did observe a higher prevalence of MetS and less favorable lipid and glucose profiles among the participants taking PIs, compared to the ones on non-PI based ART.

Of all lipid and non-lipid markers of CVD, the greatest difference, with a moderate trend toward significance, was observed for HDL-C. The individuals without MetS had on average 7 mg/dl higher HDL-C, compared to those with MetS. Knowing that for every 1 mg/dl increase in HDL-C, CVD risk is reduced by 2-3% [19], this near statistical significance should not be ignored as potentially clinically relevant. It is also worth noting that none of our participants received nevirapine, a non-nucleoside reverse transcriptase inhibitor drug known to increase HDL-C levels [20].

The American Diabetes Association (ADA) has recently recommended the use of HbA1c as one of the diagnostic tests for type 2 diabetes and also suggests a range of 5.7% to 6.4% as a category of increased risk for diabetes (i.e., pre-diabetes) [21]. Additionally, most epidemiological studies have shown that HbA1c, unlike FG, is an independent risk factor for CVD [21,22]. According to the ADA standards, the overall average HbA1c levels for both of our groups (5.7% and 6.7%) can be identified as pre-diabetes risk, while the MetS group, compared to those without MetS, may even possess a greater risk for cardiometabolic abnormalities. Hemoglobin A1c has rarely been examined in PLWH in relation to MetS, while in our cohort and among all traditional risk factors, including FG and homeostasis model assessment, it represents the strongest predictor for MetS. Thus, our findings suggest that HbA1c might be a useful diagnostic criterion for MetS in this patient population.

Many factors, such as a sedentary lifestyle and excessive caloric intake, contribute to the incidence of MetS [23]. We did not observe any differences between the groups on anthropometric measurements and levels of physical fitness, while caloric intake and four variables related to carbohydrate consumption (total carbohydrate, total sugar, added sugar, and GL) significantly differed between the groups. Total carbohydrate intake and GL have been associated with MetS in the general population [24,25], while (added) sugars have been linked with decreased intake of essential micronutrients [26] and are hypothesized to contribute to weight gain [27]. For a similar body size, individuals with MetS consumed on average more than 600 calories per day, than those without MetS.

A significant portion of excessive caloric intake in the MetS group came from carbohydrates and more specifically from the combination of naturally occurring sugar and added artificial sugar to food and beverages. A World Health Organization report from 2002 recommended that sugars be limited to less than 10% of total energy per day. Their new draft guidelines are proposing a reduction to below 5% of total daily energy intake for additional benefits [28]. The average intake of sugar for both of our groups is substantially greater (>20% of total daily energy intake) than the recommendations, contributing together with high levels of HbA1c, to the presence of MetS. Additionally, the Dietary Guidelines for Americans recommend limiting total intake of discretionary calories, including both added sugar and solid fats, to 5%-15% per day [29]. In our study, both groups consumed added sugar alone more than the recommended range for added sugar and solid fats combined. More specifically, the individuals with MetS consumed approximately 19% (equivalent to approximately 27 teaspoons) of daily calories from added sugar, compared to 15% (16 teaspoons) among the ones without MetS. In summary, our study indicates that unhealthy dietary habits consisting of higher intake of carbohydrates in the form of sugars and resulting in chronic hyperglycemia may contribute to a higher rate of MetS in our cohort.

Lastly, our program participants were of predominantly low SES, and two-thirds reported having only completed high school or less, earning less than $15,000 per year, and even more were unemployed. Low SES has been independently associated with chronic conditions and diseases including MetS [30], but the same finding was not observed in our cohort. However, due to a small number of our participants with an average or above average income, these results should be interpreted with caution. Future studies should explore the potential influence of (low) SES on MetS among PLWH.

Strengths of the current study included a program population with extensive information obtained on metabolic risk factors, including physical fitness levels and dietary habits as lifestyle characteristics highly associated with MetS. Additionally, selecting a population of PLWH who are predominantly minority and low SES provided an opportunity for them to participate in health-promotion activities not typically available as part of their conventional treatment plan. Whereas several other protocols utilized a 6–8 hour fast before blood sampling, which could overestimate TGs and MetS rates, we utilized a 12-hour fast, thus avoiding exaggerated TGs and ensuring that serum chylomicrons would be eliminated. If participants consumed any solid or liquid calories on the morning of blood draw, they were re-scheduled for the following day and told to adhere to the 12-hour fast.

Our results are hindered by several limitations. Our study design is observational and cross-sectional, which precludes causality between changes in risk factors and the rate of MetS. All of our investigators were equally trained in performing assessments. Regardless, it was not expected that all measurements (e.g., WC and BP) would always be conducted in a uniform manner. We utilized the Omron automatic oscillometric device and not a mercury sphygmomanometer, the gold standard in obtaining BP. Nonetheless, a recent study reported a high correlation between Omron and mercury measurements (SBP: r = 0.92 and DBP: r = 0.79) and low mean between-device differences (SBP: −1.6 mmHg and DBP: −0.6 mmHg, p < 0.05 for both) [31]. The same study reported that Omron underestimates high BP, which could only result in lower overall estimates of MetS in our analysis. In our research, dietary assessment was based on a short instrument and as such more prone to error in its estimate of true dietary intake. Lastly, we did not obtain HIV RNA levels, CD4 cell counts, and the duration of exposure to ART to further examine the association between HIV specific risk factors and MetS in our cohort.

## Conclusions

In conclusion, the overall prevalence of MetS in our PLWH cohort receiving ART and of predominantly low SES is higher than previously reported in the general population and in other PLWH cohorts. Metabolic syndrome as currently described does not have a common underlying pathology. Besides genetic and environmental factors, the complexity of MetS pathology among PLWH additionally includes HIV infection, specific drugs, classes of drugs, and their associated adverse effects. Our study also shows that HbA1c has the highest association with MetS in this patient population. Furthermore, PLWH consuming greater amount of carbohydrates in the form of sugars could be more at risk for MetS. Further studies are needed to examine the relationship between risk factors and prevalence of MetS over time. Assessing physical activity and physical fitness levels and dietary habits, often omitted in the typical analysis related to HIV and MetS, should be included in future work to elucidate whether MetS is a more disease dependent or lifestyle dependent constellation of symptoms in PLWH.

## Abbreviations

MetS: Metabolic syndrome
PLWH: People living with HIV
SES: Socio-economic status
HbA1c: Hemoglobin A1c
CVD: Cardiovascular disease
NCEP ATPIII: National Cholesterol Education Program/Adult Treatment Panel III
HDL-C: High-density lipoprotein cholesterol
NHANES: National Health and Nutrition Examination Survey
ART: Antiretroviral therapy
BMI: Body mass index
WC: Waist circumference
BP: Blood pressure
VO_2max_: Maximal oxygen consumption
1-RM: One-repetition maximum
T-Chol: Total cholesterol
LDL-C: Low-density lipoprotein cholesterol
VLDL-C: Very-low density lipoprotein cholesterol
TGs: Triglycerides
FG: Fasting glucose
CRP: C-reactive protein
SBP: Systolic blood pressure
DBP: Diastolic blood pressure
CVs: Coefficients of variation
GL: Glycemic load
GI: Glycemic index
Cm: Centimeters
PIs: Protease inhibitors
CI: Confidence interval
ADA: American Diabetes Association
